# American Institute of Homœopathy—Forty-fourth Annual Session

**Published:** 1891-08

**Authors:** 


### AMERICAN INSTITUTE OF HOMŒOPATHY, FORTY-FOURTH ANNUAL SESSION.

(From the *Public Ledger.*)

The forty-fourth annual session of the American Institute of Homœopathy convened at the United States Hotel, Atlantic City, New Jersey, Tuesday morning, June 16th, Dr. Theodore Y. Kinne, the President of the Institute, occupied the chair, and the other officers present were: Vice-President, James H. McClelland, of Pittsburgh; Assistant Treasurer, Dr. T. F. Smith, of New York; General Secretary, Dr. Pemberton Dudley, of Philadelphia, and Provisional Secretary, Dr. T. M. Strong, of Macon, Georgia.

Soon after ten o'clock the Institute was called to order in the dancing-hall of the hotel by the President, and the invocation was made by Rev. Dr. Ackman, pastor of the First Presbyterian Church.

On the platform were the following ex-Presidents of the Institute: Dr. W. H. Holcombe, of New Orleans; Dr. F. H. Orme, Atlanta; Dr. J. P. Dake, Nashville; Dr. A. C. Cowperthwaite, Iowa City; Dr. A. R. Wright, Buffalo; Dr. J. C. Sanders, Cleveland; Dr. B. W. James, Philadelphia; Dr. H. D. Paine, Albany; Dr. D. H. Beckwith, Cleveland; Dr. I. T. Talbot, Boston, and Dr. J. C. Burgher, of Pittsburgh.

After the acceptance of the report of the Committee on Programme and Business, read by Dr. A. R. Wright, of Buffalo, the Institute proceeded in accordance therewith.

Dr. T. F. Smith, of New York, the Assistant Treasurer, submitted the Treasurer's report. It made the following exhibit: Receipts, including $756.31 balance from last year, $5,228.91; disbursements, $4,579.25; balance, $722.76. For cyclopædia account, $536 was reported as received and expended.

The report was referred to a committee of auditors, comprising Drs. Crank, Monroe, and Edmonson.

Dr. Pemberton Dudley, the General Secretary, read the report of the Executive Committee, stating that the original engraving of certificates of membership had been destroyed in the fire at Seventh and Cherry Streets, Philadelphia, and that a new one had been made. Also that the date for the present meeting had been fixed by the Committee.

The Publication Committee reported the printing of the proceedings of 1890 in a volume of eight hundred and fifty-seven octavo pages and the distribution of the same.

The programme of business for the International Convention was reported by Dr. A. R. Wright, of Buffalo, from the committee appointed for that purpose, and it was adopted.

Dr. Millie J. Chapman, of Pittsburgh, reported a deficiency in the members of the Board of Censors present, which was supplied by the appointment of Drs. S. R. Beckwith and C. J. Higbee.

### PROGRESS IN FOREIGN COUNTRIES.

Dr. Eugene F. Storke, of Denver, Chairman of the Committee on Foreign Correspondence, made a report, which was frequently interrupted with applause.

A vote of thanks was tendered Dr. Storke, and it was referred to the Committee on Publication.

Dr. J. P. Dake, of Tennessee, presented the report of the Committee on International Pharmacopœia. The Committee are progressing in their work, and the volume will soon be ready for the printer.

### INSURANCE DISCRIMINATION.

Dr. A. C. Cowperthwaite, Chairman of the Committee on Life Insurance Examiners, presented a supplemental report, which was received with marked interest. He stated that, as Chairman of the Committee, he had written a personal letter to the President of each life insurance company that had failed to respond to the circular letter mentioned in the report of 1890, twenty-seven in number. Last year he stated that he had corresponded with all the life insurance companies of the United States. Most of the companies replied in a prompt and courteous manner, and some did not, which was reported last June at the Institute meeting. The Committee was continued and instructed to secure replies from all who had previously failed to answer. Of the twenty-seven companies he had received word from eleven, and sixteen had entirely ignored the request. The companies replying are Ætna, of New York; Maryland Life, of Baltimore; Mutual Life, of Louisville, Ky.; National Life, of Montpelier, Vt.; New York Life, of New York; Pacific Mutual Life, of San Francisco; Provident Life and Trust Company, of Philadelphia; Prudential of America, at Newark; Union Mutual, of Portland, Maine.

Each of the above claim to make no discrimination against homœopathic physicians as examiners, yet some make the claim by inference rather than by plain statement. Of the latter number, and notably, the Connecticut Mutual and Union Mutual. For instance, the last-named writes: "The subject-matter of your letter has been thoroughly considered in the past, and it will be in the future, by the company, and its business will be conducted in the future as in the past, on strictly business principles."

The doctor went on to say that he was convinced that some companies have reported that they make no discrimination against homœopathic physicians, when, in point, of fact, they do, and it is understood from those who select examiners that a homœopath is never selected when an allopath can be.

The Ætna Company writes a letter stating that "they have no intention of discriminating in the appointment of medical examiners. Years ago examiners were required to be of the old school, for the reason that they were the most numerous, convenient, and best qualified (these conditions have to some extent changed). It is not the duty or intention of the company to uphold one or another theory, and it asks for a list of the homœopathic medical institutions whose certificates would be considered a fair recommendation for employment of graduates."

That they do not appoint them is attested by the traveling agent in Iowa, who positively gave the assurance that he must not appoint homœopathic examiners, and that such examinations, when made, were not accepted, was proven by Dr. S. W. S. Dinsmore, of Sharpsburg, Pa., where a homœopathic physician would have been appointed had there not been positive rules to the contrary.

Another case is the Massachusetts Mutual. There the special agent said: "We appoint regular physicians because they are the best educated."

The Penn Mutual and Union Central answered that the officer who was competent to answer the query was away, and the Committee presume his absence still continues.

Those entirely failing to reply are: Covenant Mutual and Genesee, of St. Louis; Germania and Metropolitan, of New York; Mutual Benefit, Newark; New England Mutual, Boston; N. W. Mutual, Milwaukee; Phœnix, of Hartford; State, of Worcester; United States, of New York; Vermont, of Burlington; Vermont and Washington, of New York.

The report was accepted, and on motion of Dr. Bushrod W. James, of Philadelphia, the committee was ordered to continue its investigation of the status of the companies regarding the matter.

The next business in order was the selection of a place for the next meeting of the Institute, and the following places were named: Old Point Comfort, Newport, Denver, Cape May, Chautauqua, and Richfield Springs. The first ballot resulted in no choice, and, a second ballot being taken, Washington, D. C., was selected by a majority vote.

### GENERAL STATISTICS.

Dr. T. Franklin Smith, of New York, Chairman of the Bureau of Organization, Registration, and Statistics, has prepared a report, from which it is ascertained that in the United States there are three National homœopathic societies, two sectional societies, 28 State societies, 86 local, and 19 medical clubs. In the country 40 general homœopathic hospitals are maintained, and 35 termed special hospitals. Reports have been received from 33 general and 26 special—59 in all—and in this number there are 4,604 beds. The total number of patients treated during the year was 33,169. Of this number 25,382 have been cured, 3,173 relieved, 1,009 deceased, the death-rate being 3.12. In the hospitals 3,605 patients still remain. The homœopathic dispensaries number 47 all told, from which the committee have received reports from 35, and they report having treated 109,874 patients, and made up 301,318 prescriptions; outside visits reported number 33,756. Dr. Smith likewise reports that there are 26 journals published in the interest of Homœopathy in the United States.

Dr. James H. McClelland submitted a report embodying a new set of rules for the appointment and regulation of a Committee of the Institute upon a reconstruction of medical legislation to prevent allopathic oppression.

SESSION OF WEDNESDAY, JUNE 17TH.

At 9.30 Wednesday morning a half-hour session of the American Institute of Homœopathy was held, and a general report of the Board of Censors recommending the admission of 150 members was received.

The report of the Special Committee on Reconstruction of the Legislative Committee presented Tuesday morning and the substitute offered were, upon motion of Dr. Lewis, of Buffalo, N. Y., referred to a special committee of eighteen members, as follows: New York—Couch, Paine, Schley, Moffatt, Wright, Terry, Lee, Lewis; Georgia—Orme; Ohio—Gann; Massachusetts—Talbot; Texas—Fisher; Tennessee—Dake; Pennsylvania—McClellan; Connecticut—Wilson; Louisiana—Holcombe; Michigan—Gutchell; Iowa—Cowperthwaite. The committee is to report on Friday. After this the Institute, over which Dr. Kinne, of Patterson, had presided, adjourned until Thursday morning at 9.30.

SESSION OF THURSDAY, JUNE 18TH.

As usual the American Institute of Homœopathy had its half-hour session from 9.30 to 10, and Dr. McClelland, of Pittsburgh, reported favorably on behalf of the committee appointed to formulate an expression on the completion of the

Cyclopædia of Drug Pathogenesy, including the appendix, and the Institute makes itself responsible for 400 copies. Only the indexing now remains to be done.

The report of the Necrologist was referred to a committee consisting of Dr. Bushrod W. James, of Philadelphia, and Dr. John E. Sawyer, of Minnesota, who arranged for a memorial service for the following Sunday evening.

The resignation of Dr. William H. White, a corresponding member, resident in Vienna, was read and accepted, whereupon the Institute adjourned.

### SESSION OF FRIDAY, JUNE 19TH.

At the meeting of the American Institute of Homœopathy, which marked the commencement of the fourth day's session, the Board of Censors reported on the application of a number of new members.

The Committee on Reconstruction of Medical Legislation offered the following resolutions:

"*Resolved*, That the American Institute of Homœopathy, though of unmistakable record as to class legislation and on the subject of higher medical education, deems it wise to renew its declarations of hostility to the State Board examining system, especially the single board system, as affording an opportunity for unjust discrimination.

"*Resolved*, That, as consistent with this declaration, it instructs its Committee on Medical Legislation to co-operate with the proper authorities in the several States in antagonizing this system by assisting, when necessary, to secure separate Boards.

"*Resolved*, That one hundred dollars is hereby appropriated for the incidental expenses incurred thereby."

Dr. Alexander von Villers, of Dresden, editor of the oldest homœopathic journal in existence, was elected a corresponding member.

A paper was read from the Eclectic Society, of Connecticut, relating to a medical head in the Cabinet of the United States Government, and protesting against such a course. The matter was referred to the Committee of Medical Legislation.

Dr. Sherman, of Milwaukee, spoke of a uniform method of making and marking homœopathic drugs. He is the leading pharmacist of the Northwest. He also said that the Institute has a way of overlooking oldest facts, as, for instance, tincture and materia medica.

The Institute half-hour expiring, the Congress resumed its sessions.

SESSION OF SATURDAY, JUNE 20TH.

At the meeting of the American Institute of Homœopathy yesterday, the Board of Censors reported a large number of new applications for membership, making the total admitted at the session two hundred and nine, the largest number received at any session.

Dr. C. J. Higbee, of St. Paul, was appointed Chairman of the Committee on Medical Legislation; Dr. C. E. Fisher, of San Antonio, Tex., on Medical Education. The Committee on Medical Literature was also appointed. It comprises Doctors Buck, of Cincinnati; Dello, of New York; Villers, of Dresden; Burgher, of Pittsburgh, and Kraft, of Cleveland. On Foreign Correspondence, Drs. Strong, of Macon, Ga.; Arnulphy, Chicago; Cowl, New York, and Storke, of Denver.

